# Harmine and Harmaline Downregulate TCDD-Induced Cyp1a1 in the Livers and Lungs of C57BL/6 Mice

**DOI:** 10.1155/2013/258095

**Published:** 2012-12-20

**Authors:** Mohamed A. M. El Gendy, Ayman O. S. El-Kadi

**Affiliations:** Faculty of Pharmacy and Pharmaceutical Sciences, University of Alberta, Edmonton, AB, Canada T6G 2E1

## Abstract

We previously demonstrated that *Peganum harmala* L. extract and its main active constituents, harmine and harmaline inhibit the 2,3,7,8-tetrachlorodibenzo-*p*-dioxin (TCDD)-mediated induction of the carcinogen-activating enzyme, Cyp1a1, *in vitro*. However, the effect of both alkaloids on Cyp1a1 *in vivo* has not been investigated. Therefore, the aim of this study is to examine the effect of harmine and harmaline on TCDD-mediated induction of Cyp1a1 in mice livers and lungs. C57BL/6 male mice were distributed into four groups (*n* = 6). First group received vehicle, while the second group received TCDD (i.p.). The third and fourth groups received either harmine or harmaline (i.p.) × 3 times along with TCDD one time with the mid dose of harmine and harmaline. All mice were sacrificed after 14 h from TCDD injection, and livers and lungs were isolated. The effect of harmine and harmaline on TCDD-mediated induction of Cyp1a1 mRNA, protein, and activity levels was determined using real-time PCR, Western blot analysis, and 7-ethoxyresurofin as a substrate, respectively. Our results showed that harmine and harmaline significantly decreased the TCDD-mediated induction of Cyp1a1 in both the livers and lungs. We concluded that harmine and harmaline are promising candidate to inhibit TCDD-mediated induction of Cyp1a1 in mice hepatic and extrahepatic tissues.

## 1. Introduction 

2,3,7,8-Tetrachlorodibenzo-*p*-dioxin (TCDD) is a widely distributed environmental contaminant that possesses multiple species- and tissue-specific adverse effects such as tumor promotion, teratogenicity, and immune-, hepato-, cardio- and skin toxicity. TCDD is a known carcinogen to animals and humans, and it was classified as a human carcinogen (Group 1) by International Agency of Research on Cancer (IARC) since 1997. In 2009, IARC confirmed that TCDD is a human carcinogen that is correlated to the increased mortality from all types of human cancers combined [1].

The adverse effects of TCDD are mainly mediated through binding and activation of ubiquitous transcription factor called aryl hydrocarbon receptor (AhR). AhR is found inactive in the cytoplasm until it binds to its ligand such as TCDD. Upon ligand binding, AhR gets activated and translocated to the nucleus where it heterodimerizes with another protein called AhR nuclear translocator (ARNT). The formed complex binds to its cognate DNA sequence that is found upstream of several AhR-regulated genes including CYP1A1 [2]. 

CYP1A1 is a carcinogen-activating enzyme that catalyzes the metabolic activation of several procarcinogens to their ultimate carcinogenic forms. The activity of CYP1A1 has been correlated not only to the exposure to several environmental contaminants such as polycyclic aromatic hydrocarbons (PAHs) but also to the activation of several pro-carcinogenic agents [3]. Moreover, several studies have demonstrated the positive relationship between the induction of CYP1A1 and the incidence of several human cancers such as lung, colon, and rectal cancers [4, 5]. Additionally, it has been demonstrated that the inhibition of AhR activity and its regulated gene, CYP1A1, could result in the prevention of toxic effects caused by the AhR ligands, including carcinogenicity [6].

**Figure 1:**
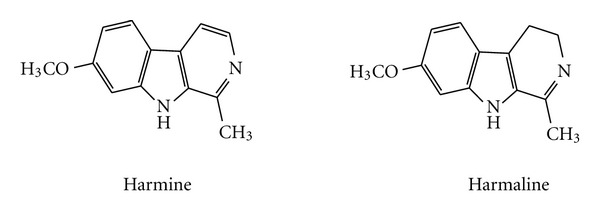
Chemical structure of harmine (7-methoxy-1-methyl-9H-pyrido [3,4-b]indole) and harmaline (4,9-dihydro-7-methoxy-1-methyl-3H-pyrido [3,4-b]indole).

Harmine, 7-methoxy-1-methyl-9H-pyrido (3,4-b)indole and harmaline, 4,9-dihydro-7-methoxy-1-methyl-3H-pyrido (3,4-b)indole (Figure 1) are common  *β*-carboline alkaloids that are present in several plants such as *Peganum harmala L*. (Nitrariaceae). Harmine and harmaline possess several pharmacological effects such as hypothermic, antimicrobial, antioxidant, hallucinogenic, cytotoxic, and antitumor properties [7]. We previously demonstrated that *Peganum harmala *extract and its main active constituents, harmine and harmaline decreased the TCDD-mediated induction of Cyp1a1 activity in mouse hepatoma Hepa-1c1c7 cells [8]. Moreover, we reported that both harmine and harmaline inhibited the TCDD-mediated induction of the carcinogen-activating enzyme, CYP1A1 in human hepatoma HepG2 cells through transcriptional and posttranslational mechanisms [9, 10]. However, the effect of both alkaloids on Cyp1a1 *in vivo *remains to be examined. Therefore, the aim of this study is to examine the effect of harmine and harmaline on TCDD-mediated induction of Cyp1a1 in mice livers and lungs.

## 2. Material and Methods

### 2.1. Chemicals and Reagents

Harmine hydrochloride (>98% pure) and 7-ethoxyresorufin (7ER) were purchased from Sigma-Aldrich (St. Louis, MO). TRIzol was obtained from Invitrogen (Carlsbad, CA). Primary antimouse Cyp1a antibody, primary rabbit antimouse actin and goat anti-rabbit IgG peroxidase secondary antibodies were purchased from Santa Cruz (Santa Cruz, CA). TCDD, >99% pure, was obtained from Cambridge Isotope Laboratories (Woburn, MA). Harmaline hydrochloride dihydrate (>90% pure) was supplied by ACROS Organics (Morris Plains, NJ). Goat antimouse IgG peroxidase secondary antibody was purchased from R&D Systems (Minneapolis, MN). High-Capacity cDNA Reverse Transcription Kit and SYBR Green PCR Master Mix were obtained from Applied Biosystems (Foster City, CA). Nitrocellulose membranes were purchased from Bio-Rad (Hercules, CA). Chemiluminescence Western blotting detection reagents were obtained from GE Healthcare Life Sciences (Piscataway, NJ). Primers were purchased from Integrated DNA technologies (IDT, Coralville, IA). All other chemicals were purchased from Fisher Scientific (Toronto, ON).

### 2.2. Animals

All experimental procedures involving animals were approved by the University of Alberta Health Sciences Animal Policy and Welfare Committee. Twenty-four male C57BL/6 mice weighing 20–25 g were obtained from Charles River Canada (St. Constant, QC, Canada). All animals were exposed to 12 h light/dark cycles and were allowed free access to food and water.

### 2.3. Mice Treatment and Tissues Isolation

Twenty-four male C57BL/6 mice were divided randomly into four groups (*n* = 6). The first group served as weight-matched controls and received the same volume of vehicle for the indicated time points. The second group were treated intraperitoneally (i.p.) with TCDD (15 *μ*g/kg) dissolved in corn oil at 0 h. The third and fourth groups were treated either with harmine hydrochloride or harmaline hydrochloride (10 mg/kg, i.p.) dissolved in normal saline with sonication and heating for 30 minutes at 40°C and TCDD (15 *μ*g/kg, i.p.). Harmine hydrochloride and harmaline hydrochloride were administered to mice at −4, 0, and +4 h, while TCDD was treated once at 0 h (Table 1). All animals were sacrificed by cervical dislocation after +14 h from TCDD treatment. Liver and lung tissues were excised and divided in two separate parts; one smaller part was kept for total RNA isolation and the larger part was used for microsomal fraction isolation, immediately frozen in liquid nitrogen and stored at −80°C until analysis.

### 2.4. RNA Isolation and cDNA Synthesis

The total RNA was isolated from frozen tissues using TRIzol reagent, according to the manufacturer's instructions (Invitrogen) and quantified by measuring the absorbance at 260 nm. RNA quality was determined by measuring the 260/280 ratio, and the first strand cDNA was synthesized using the High-Capacity cDNA Reverse Transcription Kit (Applied Biosystems) according to the manufacturer's instructions. Briefly, 1.5 *μ*g of total RNA from each sample was added to a mix of 2.0 *μ*L of 10X reverse transcriptase buffer, 0.8 *μ*L of 25X dNTP mix (100 mM), 2.0 *μ*L of 10X reverse transcriptase random primers, 1.0 *μ*L of MultiScribe reverse transcriptase, and 4.2 *μ*L of nuclease-free water. The final reaction mix was kept at 25°C for 10 min, heated to 37°C for 120 min, heated for 85°C for 5 s, and finally cooled to 4°C [11].

### 2.5. Quantification of mRNA Expression by Real-Time Polymerase Chain Reaction (Real-Time PCR)

Real-time PCR reactions were performed using an ABI 7500 system (Applied Biosystems, Inc., Foster City, CA) as described previously [12]. The primers used in the current study were chosen from previously published studies [8, 13] and were purchased from Integrated DNA Technologies (IDT, Coralville, IA). Mouse Cyp1a1: forward primer 5′-GGT TAA CCA TGA CCG GGA ACT-3′, reverse primer 5′-TGC CCA AAC CAA AGA GAG TGA-3′, and mouse 18S: forward primer 5′-GTA ACC CGT TGA ACC CCA TT-3′, reverse primer 5′-CCA TCC AAT CGG TAG TAG CG-3′. Assay controls were incorporated onto the same plate, namely, no-template controls to test for the contamination of any assay reagents. The real-time PCR data were analyzed using the relative gene expression (ΔΔCt) method, as described in Applied Biosystems User Bulletin No. 2 [14].

### 2.6. Microsomal Fraction Isolation and Western Blot Analysis

Liver and lung microsomal fractions were isolated as described previously [15]. Briefly, frozen tissues were cut into small pieces and homogenized separately in cold sucrose solution (1 g of tissue in 5 mL of 0.25 M sucrose). Thereafter, the microsomal fractions were separated using differential ultracentrifugation process. The resulted microsomal pellets were reconstituted in ice-cold sucrose solution and stored at −80°C till used. Protein content of each microsomal fraction was determined by Lowry method using bovine serum albumin as a standard [16]. Western blot analysis was performed as described previously [8]. Briefly, microsomal proteins (2 *μ*g) were loaded onto a 10% SDS-PAGE and electrophoretically transferred to a nitrocellulose membrane. The protein blots were blocked for 24 h at 4°C in blocking buffer (5% skim milk powder, 2% bovine serum albumin, and 0.05% (v/v) Tween 20 in Tris-buffered saline solution (0.15 M sodium chloride, 3 mM potassium chloride, and 25 mM Tris base)). Thereafter, the protein blots were incubated with primary antimouse Cyp1a antibody for 2 h at room temperature or primary rabbit antimouse actin for 24 h at 4°C. Finally, the membranes were incubated with peroxidase-conjugated secondary antibodies for another 1 h, namely, goat antimouse IgG for Cyp1a, or goat anti-rabbit IgG for actin detection. The formed bands were visualized with the enhanced chemiluminescence method according to the manufacturer's instructions (GE Healthcare, Piscataway, NJ). The intensity of protein bands was quantified relative to the signals obtained for actin protein using Java-based image-processing software, ImageJ (W. Rasband (2005) National Institutes of Health, Bethesda, MD, http://rsb.info.nih.gov/ij/).

### 2.7. Microsomal Incubation and Determination of Cyp1a1 Enzymatic Activity

Microsomes from Liver (0.25 mg/mL) or lung (0.15 mg/mL) were suspended in incubation buffer containing 3 mM magnesium chloride hexahydrate dissolved in 0.5 M potassium phosphate buffer pH 7.4 at 37°C in a shaking water bath (100 rpm). 7-Ethoxyresorufin was used as a substrate and its final concentration was 2 *μ*M. A pre-equilibration period of 5 min was performed before initiating the reaction with 1 mM NADPH. After an incubation period of 3 min for liver microsomes and 30 min for lung microsomes, the reaction was stopped by adding 1 mL of ice-cold methanol. The reaction was carried out in duplicate using a reaction mixture without NADPH as a blank for each microsome sample. The amount of resorufin in the supernatant of each reaction mixture was determined using the Baxter 96-well fluorescence plate reader using excitation and emission wavelengths of 545 and 575 nm, respectively. Formation of resorufin was linear with incubation time and protein amount. The amount of resorufin was calculated using a standard curve of known resorufin concentrations, and the final amount was calculated by subtracting the amount of resorufin formed in each blank from its corresponding reaction mixture. The final enzymatic activities were expressed as picomole of resorufin formed per minute and per milligram of microsomal proteins.

### 2.8. Statistical Analysis

All results are presented as mean ± S.E.M., and statistical differences between treatment groups were determined using one way ANOVA followed by the Student-Newman-Keuls post hoc test using SigmaStat 3.5 program for Windows, Systat Software Inc. (San Jose, CA).

## 3. Results

### 3.1. Effect of Harmine and Harmaline on Cyp1a1 mRNA, Protein, and Enzymatic Activity in C57BL/6 Mice Livers

**Figure 2:**
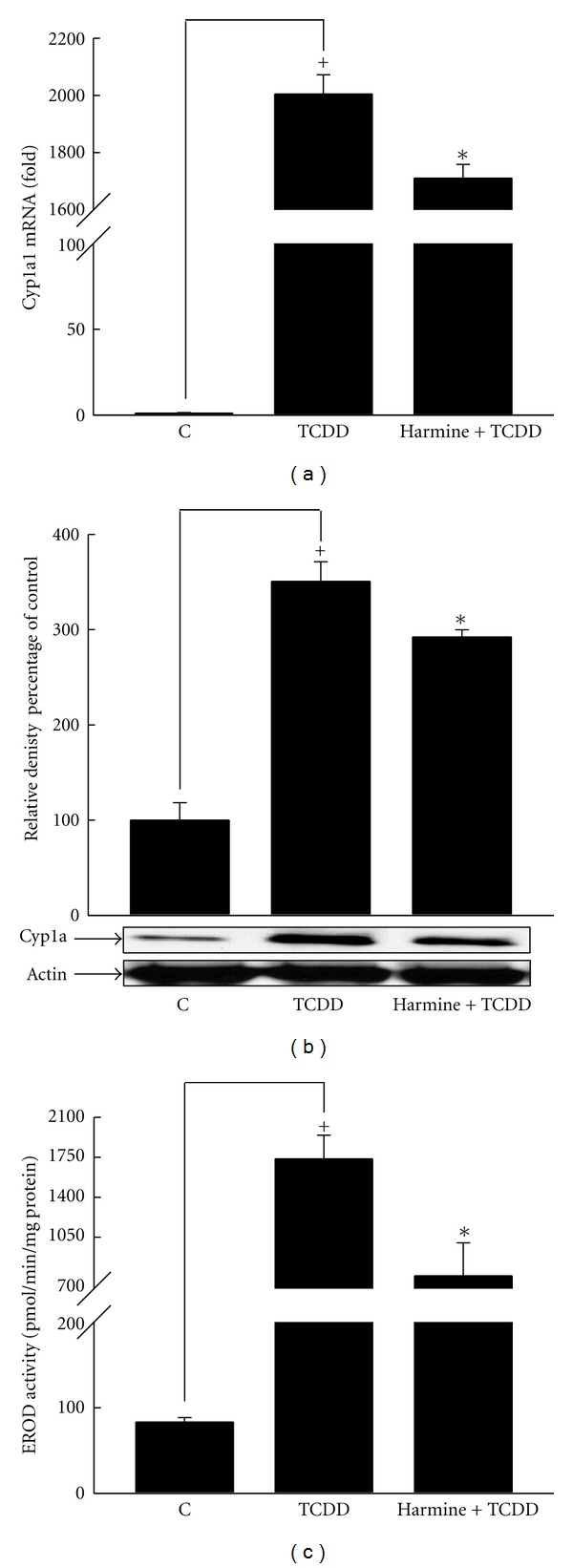
Effect of harmine on TCDD-mediated induction of hepatic Cyp1a1 at mRNA (a), protein (b), and catalytic activity (c) in C57BL/6 mice. Mice were distributed into several groups, receiving the used vehicle (weight-matched control), TCDD, or TCDD and harmine. After 14 h from TCDD treatment, mice were sacrificed, and the livers were isolated. Total RNA was isolated using TRIzol reagent, and microsomal fractions were isolated using ultracentrifugation. The level of Cyp1a1 mRNA was determined using real-time PCR (a). Furthermore, Cyp1a protein and Cyp1a1 catalytic activity were determined in microsomal fractions using Western blot analysis (b) and 7ER as a substrate (c), respectively. Values represent the mean ± S.E.M. (*n* = 6). (+) *P* < 0.05 compared to control (c), (∗) *P* < 0.05 compared to  *T*.

Our results showed that treatment of mice with TCDD significantly increased the level of Cyp1a1 mRNA by approximately 200,000% compared to the control group. Moreover, treatment with harmine significantly decreased TCDD-mediated induction of Cyp1a1 mRNA expression by 15% (Figure 2(a)). 

To examine whether the effect of harmine on hepatic Cyp1a1 mRNA is translated to a relevant effect at protein and enzymatic activity levels, microsomal fractions were isolated from livers and the effect of harmine on Cyp1a protein and Cyp1a1 enzymatic activity was determined using Western blot analysis and 7ER as a substrate, respectively. Our results showed that TCDD induced Cyp1a protein by 250% relative to the control group. On the other hand, treatment of harmine significantly decreased TCDD-mediated induction of Cyp1a protein by 17% (Figure 2(b)). Moreover, TCDD induced Cyp1a1 enzymatic activity by 2000% relative to the control group, whereas harmine treatment significantly decreased TCDD-mediated Cyp1a1-dependent enzymatic activity by 60% (Figure 2(c)).

**Figure 3:**
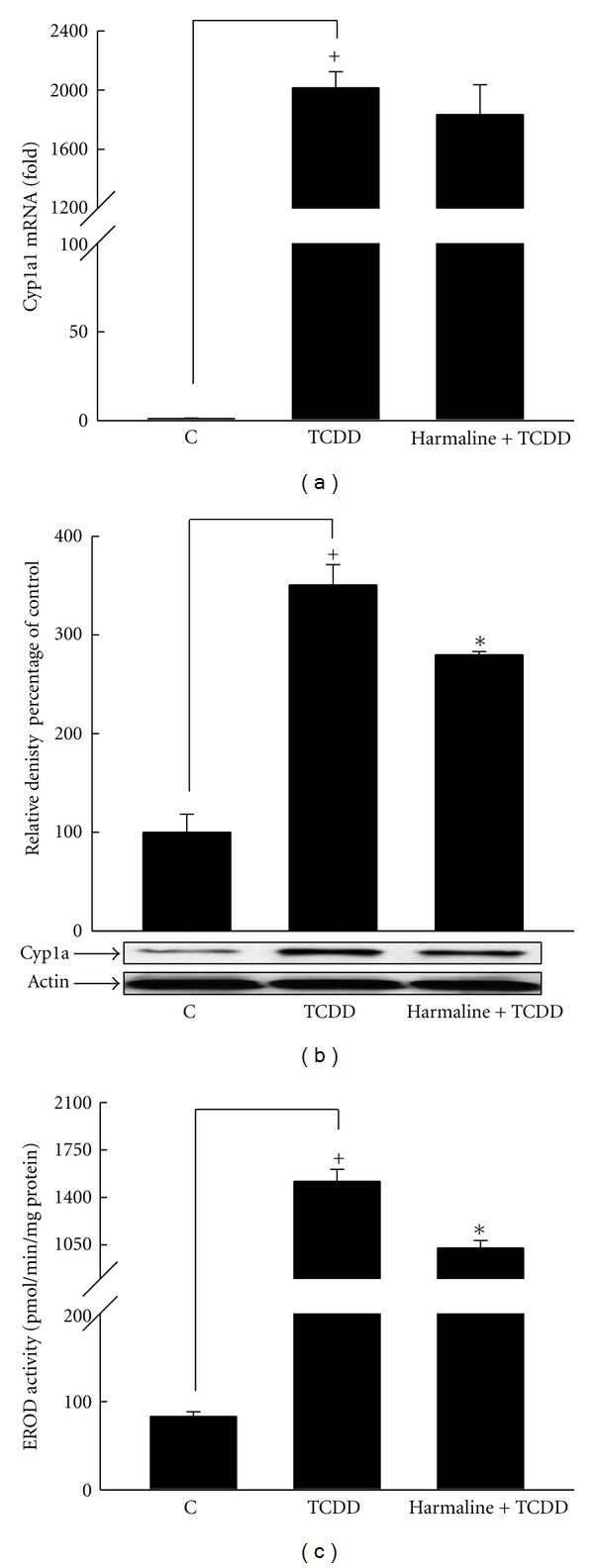
Effect of harmaline on TCDD-mediated induction of hepatic Cyp1a1 at mRNA (a), protein (b), and catalytic activity (c) in C57BL/6 mice. Mice were distributed into several groups, receiving the used vehicle (weight-matched control), TCDD, or TCDD and harmaline. After 14 h from TCDD treatment, mice were sacrificed, and the livers were isolated. Total RNA was isolated using TRIzol reagent, and microsomal proteins were isolated using ultracentrifugation. The level of Cyp1a1 mRNA was determined using real-time PCR (a). Furthermore, Cyp1a protein and Cyp1a1 catalytic activity were determined in microsomal fractions using Western blot analysis (b) and 7ER as a substrate (c), respectively. Values represent the mean ± S.E.M. (*n* = 6). (+) *P* < 0.05 compared to control (c), (∗) *P* < 0.05 compared to  *T*.

On the other hand, our results showed that harmaline decreased the level of TCDD-mediated induction of Cyp1a1 mRNA expression by 9%; however, the effect was not significant (Figure 3(a)). Furthermore, harmaline significantly decreased Cyp1a protein by 20% and Cyp1a1 enzymatic activity by 32% using Western blotting and 7ER as a substrate, respectively (Figures 3(b) and 3(c)). Collectively, both alkaloids decreased TCDD-mediated induction of Cyp1a1 in liver tissues; however, harmine showed a more pronounced effect especially at Cyp1a1 enzymatic activity level (Table 2). 

### 3.2. Effect of Harmine and Harmaline on Cyp1a1 mRNA, Protein, and Enzymatic Activity Levels in C57BL/6 Mice Lungs

**Figure 4:**
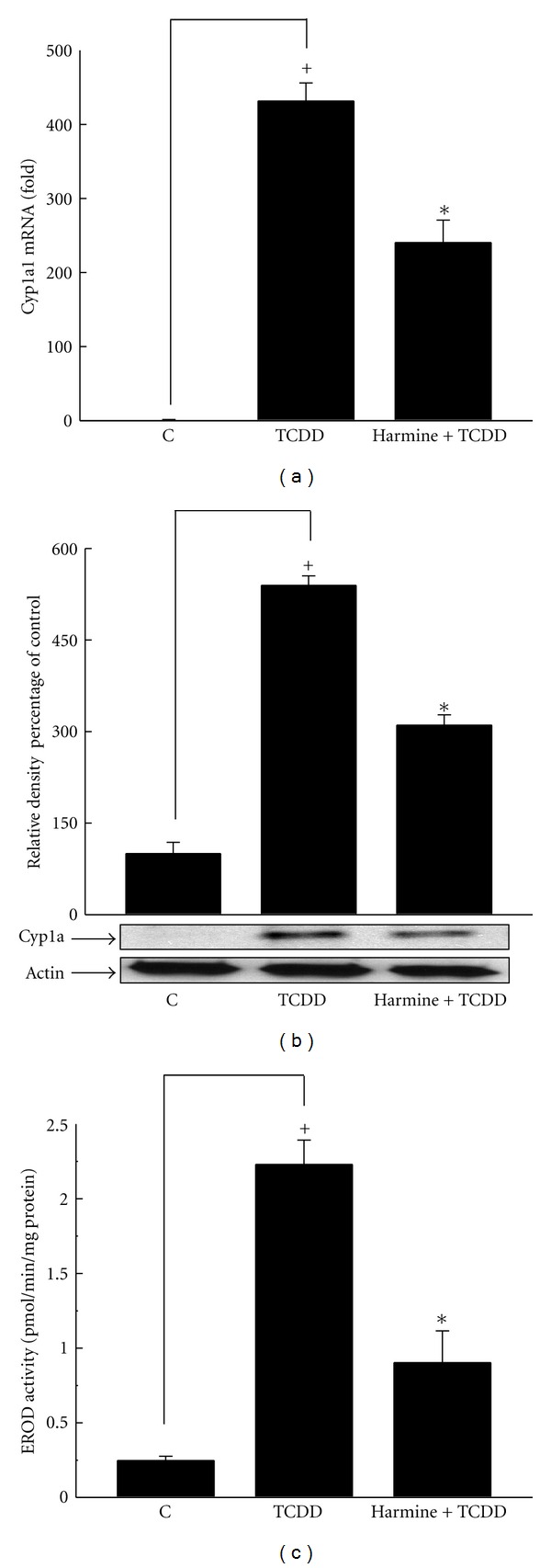
Effect of harmine on TCDD-mediated induction of lung Cyp1a1 at mRNA (a), protein (b), and catalytic activity (c) in C57BL/6 mice. Mice were distributed into several groups, receiving the used vehicle (weight-matched control), TCDD, or TCDD and harmine. After 14 h from TCDD treatment, mice were sacrificed, and the lungs were isolated. Total RNA was isolated using TRIzol reagent, and microsomal fractions were isolated using ultracentrifugation. The level of Cyp1a1 mRNA was determined using real-time PCR (a). Furthermore, Cyp1a protein and Cyp1a1 catalytic activity were determined in microsomal fractions using Western blot analysis (b) and 7ER as a substrate (c), respectively. Values represent the mean ± S.E.M. (*n* = 6). (+) *P* < 0.05 compared to control (c), (∗) *P* < 0.05 compared to  *T*.

In an effort to examine whether the effect of harmine and harmaline is not specific to liver tissues, lung tissues were isolated, and the effect of both alkaloids on TCDD-mediated induction of Cyp1a1 was determined at mRNA, protein, and enzymatic activity levels. Our results showed that TCDD significantly induced the lung Cyp1a1 mRNA expression by 43,000% compared to control group, whereas harmine significantly decreased TCDD-mediated induction of lung Cyp1a1 mRNA expression by 44% (Figure 4(a)). Moreover, TCDD caused induction of lung Cyp1a protein and Cyp1a1 enzymatic activity by 440% and 792%, respectively (Figures 4(b) and 4(c)). On the other hand, harmine significantly decreased TCDD-mediated induction of lung Cyp1a protein and enzymatic activity by 43% and 60%, respectively (Figures 4(b) and 4(c)). Taken together, these data demonstrate that the effect of harmine on TCDD-mediated Cyp1a1 is similar in liver and lung tissues especially at Cyp1a1 enzymatic activity level.

**Figure 5:**
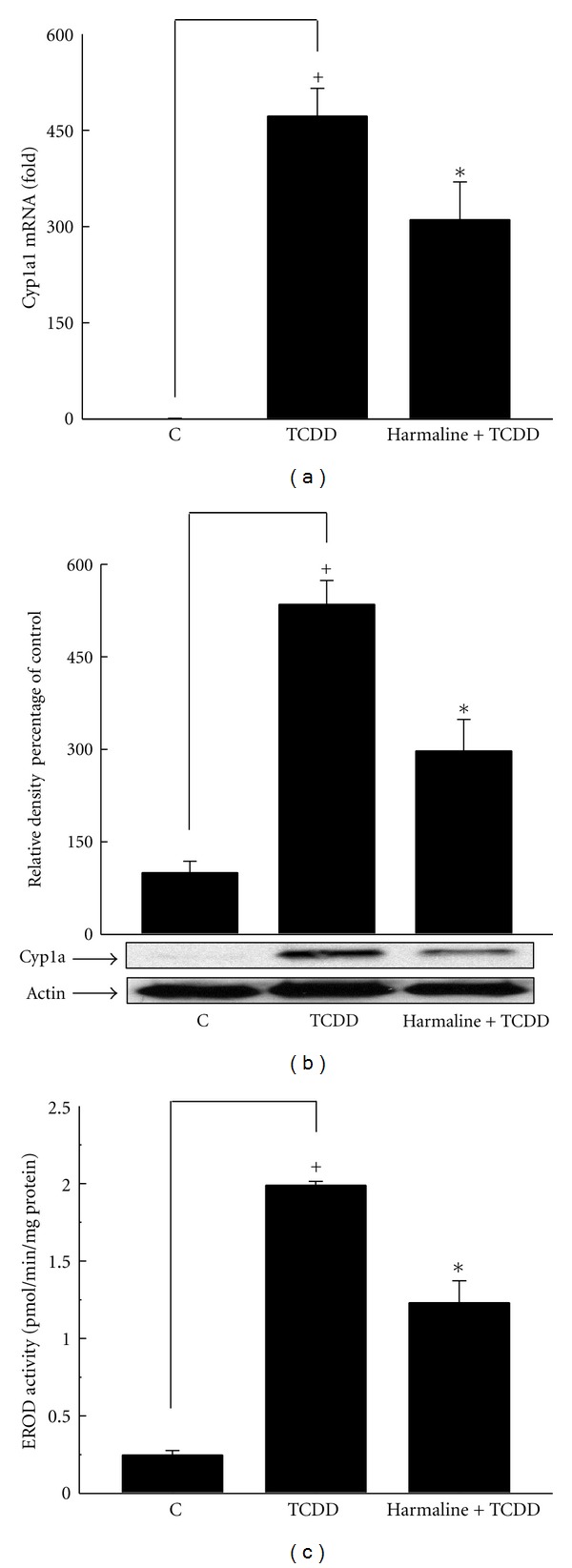
Effect of harmaline on TCDD-mediated induction of lung Cyp1a1 at mRNA (a), protein (b), and catalytic activity (c) in C57BL/6 mice. Mice were distributed into several groups, receiving the used vehicle (weight-matched control), TCDD, or TCDD and harmaline. After 14 h from TCDD treatment, mice were sacrificed, and the lungs were isolated. Total RNA was isolated using TRIzol reagent, and microsomal fractions were isolated using ultracentrifugation. The level of Cyp1a1 mRNA was determined using real-time PCR (a). Furthermore, Cyp1a protein and Cyp1a1 catalytic activity were determined in microsomal fractions using Western blot analysis (b) and 7ER as a substrate (c), respectively. Values represent the mean ± S.E.M. (*n* = 6). (+) *P* < 0.05 compared to control (c), (∗) *P* < 0.05 compared to  *T*.

Similar to harmine, we tested the effect of harmaline on TCDD-mediated induction of lung Cyp1a1 at mRNA, protein and enzymatic activity levels. Our results demonstrated that harmaline decreased TCDD-mediated induction of lung Cyp1a1 mRNA expression by 34% (Figure 5(a)), whereas, it significantly decreased TCDD-mediated Cyp1a protein and Cyp1a1 enzymatic activity by 44% and 40%, respectively (Figures 5(b) and 5(c)). Taken together, these data demonstrate that the effect of harmaline on TCDD-mediated Cyp1a1 enzymatic activity is almost the same in lung and liver tissues. Similar to liver tissue, harmine showed a more pronounced effect in decreasing TCDD-mediated induction of lung Cyp1a1 enzymatic activity than that observed with harmaline (Table 2).

## 4. Discussion

The present study demonstrates for the first time that harmine and harmaline significantly decreased the TCDD-mediated induction of the carcinogen-activating enzyme Cyp1a1 in livers and lungs of C57BL/6 mice.

Cancer development is a multistage process that involves several factors. Inherited genetic factors can explain the incidence of 5–15% of most cancers, but environment and lifestyle are the major factors contributing to cancer development [17]. TCDD is a widely distributed environmental pollutant that is usually released in the environment from several sources such as waste incinerators, ferrous and non-ferrous metal production, herbicides manufacturing, and power generation [18]. Several accidents and occupational exposures to TCDD demonstrated the role of TCDD in the increased risk of cancer incidence and mortality [1]. TCDD is a metabolically stable AhR ligand, and several adverse effects of TCDD exposure are related to the persistent activation of the AhR signaling pathway. In agreement with this hypothesis are the results of experiments using transgenic mice in which AhR function has been compromised. It has been demonstrated that the TCDD-mediated adverse effects are attenuated in mice possessing disrupted AhR function [2, 19].

Most of the chemical carcinogens in the environment are chemically inert by themselves and require metabolic activation by cytochrome P450 (CYP) enzymes to more reactive metabolites in order to exhibit carcinogenicity in experimental animals and humans [20]. It is well known that AhR regulates numerous CYP, such as CYP1A1, that participate in the metabolic activation of several procarcinogens and their conversion to ultimate carcinogenic forms [2]. Consistent with this hypothesis, it has been previously reported that TCDD induces expression of persistent high level of CYP1A1 that leads to increased metabolism of exogenous and endogenous chemicals, generation of reactive oxygen species and induce oxidative stress that results in increased DNA damage [2, 21]. Moreover, recent reports have highlighted the use of AhR as a target for new chemopreventative agents. In this context, several AhR antagonists have shown promising results against numerous carcinogenic agents. It has been previously reported that the genotoxicity associated with benzo(a)pyrene in mice was inhibited by AhR antagonists such as 3′-methoxy-4′-nitroflavone and resveratrol [22, 23].

Harmine is an aromatic  *β*-carboline compound that is structurally similar to its dihydro-*β*-carboline analogue, harmaline (Figure 1). Both compounds are found naturally in several plants such as *Peganum harmala*, and they possess several pharmacological effects including antitumor properties. Harmine and harmaline are metabolized in liver and extrahepatic tissues to their main metabolites, harmol, and harmalol, respectively, mainly by CYP2D6 and CYP1A2 (Figure 1) [24]. We have previously demonstrated that both harmine and harmaline are capable of inhibiting TCDD-mediated induction of Cyp1a1 in murine hepatoma Hepa 1c1c7 cells [8]. Moreover, we demonstrated that both compounds act as AhR antagonists. Finally, we confirmed that harmine and harmaline possess posttranslational modification by which they reduce the CYP1A1 protein stability in human hepatoma HepG2 cells [9, 10]. Therefore, we hypothesized that the effect of harmine and harmaline can be translated *in vivo* using a responsive C57BL/6 mouse strain. Moreover, we tested whether or not the effect of harmine and harmaline can be demonstrated in other extrahepatic tissues, using lung as a representative tissue.

In the current study, we have chosen the C57BL/6 mouse strain as it contains a responsive AhR allele (AhR^b^) [2]. Regarding the selection of TCDD dose, it is known that TCDD is a metabolically stable compound, and its half-life has been previously determined in mice to be around 20 days [25]. Several concentrations of TCDD have been examined previously for Cyp1a1 induction and AhR activation in the same mouse strain, and it was demonstrated that 15 *μ*g/kg (i.p.) provides a submaximal saturation/activation of the AhR [26]. Additionally, harmine and harmaline doses have been selected according to their half-lives. It has been demonstrated previously that harmine possesses a short half-life in rodents estimated to be around 20 min, whereas harmaline possesses a relatively longer half-life, around 60 min [27]. Therefore, we thought that multiple doses of both alkaloids would be advantageous. Most importantly, 10 mg/kg body weight for three doses has been selected based on preliminary experiments, in which we could not detect any effect with lower doses. On the other hand, it is well established that the major drawbacks of using these  *β*-carboline alkaloids are their tremorgenic side effects [7]. In our study, slight to moderate tremors have been detected for harmine and harmaline in the first dose with higher effect with harmaline. However, these tremors decreased dramatically in the subsequent doses.

There are several reasons behind the choice of liver tissue in our study. First, we have previously studied the effect of harmine and harmaline using different human and murine hepatoma cells. Second, it is well established that TCDD is concentrated in the body mainly in adipose tissue and liver [28]. TCDD is sequestered in the liver by liver-specific microsomal binding proteins [29]. Third, active AhR is an important factor for developing TCDD-mediated hepatocellular toxicity [30]. Finally, the liver is the place of maximum metabolism and highest amounts of CYP enzymes, with a maximum level of CYP1A1 induction. On the other hand, lung has been selected in our study because it is one of the highly exposed organs to environmental pollutants through smoking and air pollutants. Furthermore, several studies have correlated the induction of CYP1A1 enzymatic activity with the development of lung cancer [4]. 

In the current study, harmine and harmaline significantly decreased TCDD-mediated Cyp1a1 induction in mice livers and lungs. Harmine showed a greater effect than harmaline in both liver and lung tissues. The differences between the effect of harmine and harmaline can be attributed to two main reasons. First, there is a structural difference between both alkaloids. Harmine has an aromatic planar structure that can enhance its binding ability to AhR, whereas harmaline possesses a coplanar structure. In this context, we have previously demonstrated that harmine efficiently displaced radiolabeled-TCDD in a ligand competition binding assay, whereas harmaline showed a modest effect [9, 10]. Second, harmine and harmaline possess different pharmacokinetic parameters. It was estimated that the ability of harmine to concentrate in lung is more than that observed for harmaline in rodents [27].

## 5. Conclusion

Harmine and harmaline decrease the TCDD-mediated induction of the carcinogen-activating enzyme Cyp1a1 in C57BL/6 mice livers and lungs. These data provide the first evidence that harmine and harmaline can prevent the adverse effect of dioxins and other AhR ligands *in vivo*.

## Figures and Tables

**Table 1:** Representation of different groups of C57BL/6 mice and the dose schedule for each group.

Treatments groups		Time (h)		
	−4	0	+4	+14
Group 1	Saline	Saline + corn oil	Saline	sacrifice
Group 2	Saline	Saline + TCDD (15 *μ*g/kg)	Saline	sacrifice
Group 3	Harmine (10 mg/kg)	Harmine (10 mg/kg) + TCDD (15 *μ*g/kg)	Harmine (10 mg/kg)	sacrifice
Group 4	Harmaline (10 mg/kg)	Harmaline (10 mg/kg) + TCDD (15 *μ*g/kg)	Harmaline (10 mg/kg)	sacrifice

**Table 2:** Summary of the effects of harmine and harmaline on TCDD-mediated induction of Cyp1a1 in livers and lungs of C57BL/6 mice.

		Harmine	Harmaline
	TCDD-induced		
Liver	(i) mRNA Cyp1a1	↓ 15%	↓ 9%
	(ii) Protein Cyp1a	↓ 17%	↓ 20%
	(iii) Activity Cyp1a1	↓ 60%	↓ 32%

	TCDD-induced		
Lung	(i) mRNA Cyp1a1	↓ 44%	↓ 34%
	(ii) Protein Cyp1a	↓ 43%	↓ 44%
	(iii) Activity Cyp1a1	↓ 60%	↓ 40%

## Abbreviations

AhR: Aryl hydrocarbon receptor
CYP1A1: Cytochrome P450 1A1
7ER: 7-Ethoxyresorufin
TCDD: 2,3,7,8-Tetrachlorodibenzo-*p*-dioxin.
